# The Structure of the N-Terminus of Kindlin-1: A Domain Important for αIIbβ3 Integrin Activation

**DOI:** 10.1016/j.jmb.2009.09.061

**Published:** 2009-12-18

**Authors:** Benjamin T. Goult, Mohamed Bouaouina, David S. Harburger, Neil Bate, Bipin Patel, Nicholas J. Anthis, Iain D. Campbell, David A. Calderwood, Igor L. Barsukov, Gordon C. Roberts, David R. Critchley

**Affiliations:** 1 Department of Biochemistry, Henry Wellcome Building, University of Leicester, Lancaster Road, Leicester LE1 9HN, UK; 2 Department of Pharmacology and Interdepartmental Program in Vascular Biology and Transplantation, Yale University School of Medicine, New Haven, CT 06520, USA; 3 Department of Biochemistry, University of Oxford, South Parks Road, Oxford OX1 3QU, UK; 4 School of Biological Sciences, University of Liverpool, Crown Street, Liverpool L69 7ZB, UK

**Keywords:** CHO, Chinese hamster ovary, FA, focal adhesions, FACS, fluorescence-activated cell sorting, FERM, four-point-one, ezrin, radixin, moesin, F0–F3, FERM subdomains 0–3, GFP, green fluorescent protein, HSQC, heteronuclear single quantum coherence, MFI, mean fluorescence intensity, NOE, nuclear Overhauser enhancements, NOESY, nuclear Overhauser enhancement spectroscopy, PDB, Protein Data Bank, PH, pleckstrin homology, kindlin, structure, integrin, talin, focal adhesion

## Abstract

The integrin family of heterodimeric cell adhesion molecules exists in both low- and high-affinity states, and integrin activation requires binding of the talin FERM (four-point-one, ezrin, radixin, moesin) domain to membrane-proximal sequences in the β-integrin cytoplasmic domain. However, it has recently become apparent that the kindlin family of FERM domain proteins is also essential for talin-induced integrin activation. FERM domains are typically composed of F1, F2, and F3 domains, but the talin FERM domain is atypical in that it contains a large insert in F1 and is preceded by a previously unrecognized domain, F0. Initial sequence alignments showed that the kindlin FERM domain was most similar to the talin FERM domain, but the homology appeared to be restricted to the F2 and F3 domains. Based on a detailed characterization of the talin FERM domain, we have reinvestigated the sequence relationship with kindlins and now show that kindlins do indeed contain the same domain structure as the talin FERM domain. However, the kindlin F1 domain contains an even larger insert than that in talin F1 that disrupts the sequence alignment. The insert, which varies in length between different kindlins, is not conserved and, as in talin, is largely unstructured. We have determined the structure of the kindlin-1 F0 domain by NMR, which shows that it adopts the same ubiquitin-like fold as the talin F0 and F1 domains. Comparison of the kindlin-1 and talin F0 domains identifies the probable interface with the kindlin-1 F1 domain. Potential sites of interaction of kindlin F0 with other proteins are discussed, including sites that differ between kindlin-1, kindlin-2, and kindlin-3. We also demonstrate that F0 is required for the ability of kindlin-1 to support talin-induced αIIbβ3 integrin activation and for the localization of kindlin-1 to focal adhesions.

## Introduction

Cell adhesion to the extracellular matrix is fundamental to the development of multicellular organisms^1^ and involves the coordinated assembly and disassembly of the integrin family of heterodimeric (αβ) cell adhesion molecules into complexes containing cytoplasmic proteins with linker, scaffolding, adaptor, regulatory, and mechano-transduction functions.^2–5^ Studies at the cellular and organismal level show that the cytoskeletal protein talin plays a pivotal role in this regard.^6–8^ Thus, talin provides a direct link between the β-integrin subunit and the actin cytoskeleton^9–11^ and acts as a scaffold for the recruitment of other proteins such as vinculin, which is known to be important for stabilizing integrin-containing cell matrix junctions [focal adhesions (FAs)].^12^ Talin also promotes integrin clustering^13^ and is essential for switching integrins from a low- to a high-affinity state.^14,15^

**Fig. 1:**
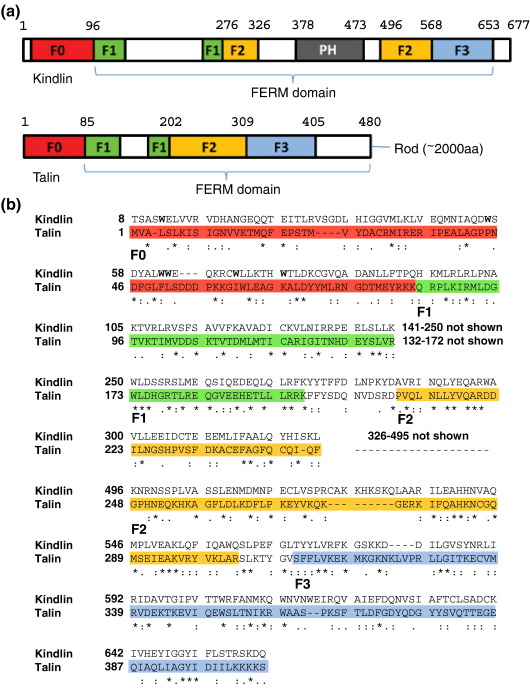
Comparison of the domain structure of kindlins and the talin head. (a) Schematic diagram of the domain structure of kindlins and talin. The individual domains—F1, F2, and F3—that make up a canonical FERM domain are shown in green, orange, and blue, respectively; the F0 domain is shown in red. The kindlin PH domain is indicated by a black box. Unstructured regions including the F1–loop region are shown in white. The position of the long C-terminal talin rod is indicated. The horizontal scale in both schematics is the same. (b) The primary sequences of mouse kindlin-1 and talin-1 FERM domains were aligned by T-Coffee. The same color scheme is used as in (a). The sequence of the F1 insert and the kindlin PH domain are not included in the alignment. The six tryptophan residues in kindlin-1 F0 are shown in bold. The overall similarity between talin-1 and kindlin-1 is relatively low (28%), due largely to the inclusion of the kindlin PH domain and the insert in F1. The similarity between the individual F0, F1, F2, and F3 domains is much higher (36–55%).

There are two talin genes in vertebrates,^16,17^ and both talin-1 and talin-2 (∼ 270 kDa; 2541 amino acids) are composed of an N-terminal head (∼ 50 kDa) and a large, flexible C-terminal rod (∼ 220 kDa).^7^ The talin head contains a FERM (four-point-one, ezrin, radixin, moesin) domain made up of F1, F2, and F3 domains but is atypical in that F1 contains a large unstructured loop and is preceded by an additional domain (F0) with homology to F1 (Fig. 1a and Goult *et al.*, under revision). The F3 FERM domain has a phosphotyrosine-binding domain fold and selectively binds to the more membrane-proximal of the two NPxY-like motifs in the short cytoplasmic tails of β-integrin subunits.^18^ The structure of talin F3 bound to residues ^739^WDTANNPLYDEA^750^ of the β3 integrin tail shows that the integrin peptide interacts predominantly with the hydrophobic surface on strand S5 of the F3 domain.^19^ More recently, F3 has also been shown to interact with the membrane-proximal helix of the β3 tail, and integrin residues F727 and F730 bind to a hydrophobic pocket in F3 made up of the flexible loop between β-strands S1 and S2.^20^ Significantly, mutation of either F727 or F730 in β3 integrin or the interacting residues in F3 (notably L325) markedly reduced activation of αIIbβ3 integrin expressed in Chinese hamster ovary (CHO) cells. This has led to a model in which talin F3 initially binds to the membrane-proximal β3 integrin NPxY motif and subsequently engages the membrane-proximal helix, breaking the clasp between the α- and β-integrin tails, which normally locks the integrin in a low-affinity state. However, the talin F3 domain alone is not sufficient to activate β1 integrins, and the F0 and F1 domains are also required.^21^

Studies on the UNC112 gene in *Caenorhabditis elegans*^22^ and the rare autosomal dominant human skin fragility disorder Kindler's syndrome^23^ indicate that the kindlin family of FERM domain proteins is also important in integrin regulation.^8,24–26^ There are three mammalian kindlin genes;^27^ kindlin-1 is highly expressed in skin and intestine, which explains why kindlin-1 mutations largely affect the skin, oral mucosa, and colon.^23,28^ Kindlin-2 is widely expressed, and kindlin-2 knockout mice show peri-implantation embryonic lethality.^29^ Kindlin-3 is only expressed in hematopoietic cells, and kindlin-3 mutations are linked to rare variants of leukocyte adhesion deficiency syndrome.^30–32^ Similarly, kindlin-3 knockout mice show bleeding disorders, and the mice die shortly after birth.^33^ Biochemical studies show that kindlin-1^34^ and kindlin-2^35^ bind to the cytoplasmic domain of β1 integrins and that this is required both to recruit kindlin-2 to FAs and for FA assembly.^35^ Interestingly, binding of kindlin-2 to β-integrin tails was independent of the membrane-proximal NPxY motif recognized by the talin F3 FERM domain. Subsequent studies showed that (i) kindlin-1, kindlin-2, and kindlin-3 bind to the membrane-distal NxxY and a preceding threonine-containing motif in integrin tails (TS^752^T and NITY^759^ in β3 integrins); (ii) kindlins act synergistically with the talin head to activate β3 integrins; and (iii) kindlins are required for talin-mediated integrin activation.^28,29,33,36^ Overall, this suggests a model in which kindlins bind to the C-terminal region of integrin tails, facilitating talin binding to more membrane-proximal sequences, either by orientating the integrin tails or by displacing other binding partners.

Kindlins are most similar in sequence to the talin FERM domain, but initial alignments suggested that this similarity was limited to the F2 and F3 domains.^34^ However, the alignments failed to take into account the large insert in the talin F1 domain and the presence of a previously unrecognized domain (F0) that precedes the talin FERM domain. We now show that kindlins have the same domain structure as the talin head and report (i) the structure of the kindlin-1 F0 domain, (ii) that this domain is essential for kindlin-1 to potentiate integrin activation by talin, and (iii) that this domain is required for localization of kindlin-1 to FAs.

## Results and Discussion

### The domain structure of kindlins

The sequences of kindlin-1 to kindlin-3 are highly conserved (Fig. S1) and are most similar to that of the talin FERM domain.^34^ The kindlins clearly contain F2 and F3 domains, although F2 contains a large insert with a pleckstrin homology (PH) domain fold [Li *et al.* unpublished results; Protein Data Bank (PDB) ID: 2YS3]. However, the original sequence alignments with talin were much less clear-cut in the N-terminal half of the sequence. Our recent structural studies on talin (Goult *et al.*, under revision) have led to the identification of (i) an ∼ 30-amino-acid insert in talin F1 and (ii) a new domain (F0) at the N-terminus of talin (Fig. 1a and Fig. S2). In light of this new information, we have reexamined the sequence alignments and have now established that kindlins also contain regions homologous to the talin F0 and F1 domains (Fig. 1 and Fig. S2), giving an overall 28% similarity between mouse kindlin-1 and talin-1.

The alignment indicates that kindlin F1 domains contain an insert in the same position as in talin-1 F1, but it is substantially larger than that in talin-1 (109 residues in kindlin-1 *versus* 40 residues in talin-1), and it varies in length between the kindlins (110–120 residues in kindlin-1 and kindlin-2 *versus* 86 residues in kindlin-3; Fig. S1). Any sequence similarity of the insert with that in talin is restricted to a short region at the C-terminal end that is predicted to have a tendency to form a helical structure. As for talin, the ^1^H-^15^N heteronuclear single quantum coherence (HSQC) spectrum of the isolated kindlin-1 insert indicates a substantially disordered conformation (Fig. S3). Both inserts contain a pair of phosphorylation sites†^37^ although the significance of this and, indeed, the function of the insert itself have not been determined. When the insert is taken into account, the sequence alignments show that the F1 domains of kindlin and talin are as similar to one another (55% similarity) as are the kindlin and talin F2 and F3 domains (50% and 54%, respectively). However, the sequence similarity between the kindlin and talin F0 domain (36%) is significantly less than that for the other domains (Fig. 1b and Fig. S2). Finally, kindlins (unlike talin) contain a region preceding the F0 domain that is variable in length between the three isoforms.

### The structure of kindlin F0

**Fig. 2:**
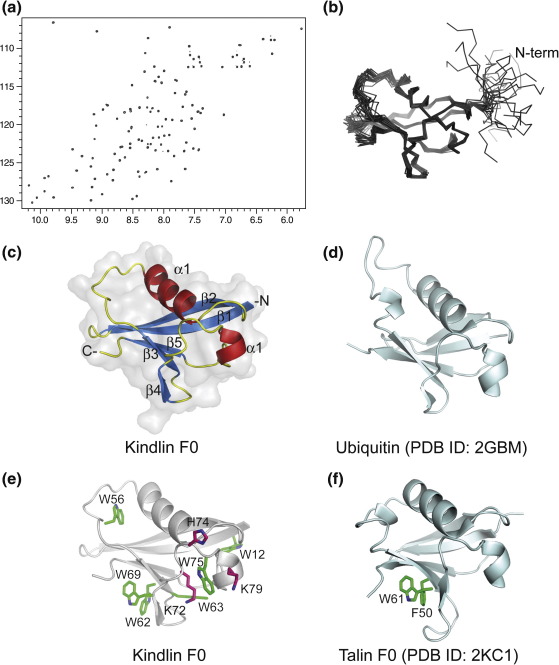
The solution structure of the F0 domain of kindlin-1. (a) ^1^H–^15^N HSQC spectrum of residues 1–96. (b) Superposition of the 20 lowest-energy solution structures of kindlin-1 F0. (c) Ribbon view of a representative low-energy structure of kindlin-1 F0 showing the β-grasp fold; secondary-structure elements are indicated. (d) The closest structural homologue of F0, ubiquitin (PDB ID: 2GBM), oriented in the same way as kindlin-1 F0 in (c), to show their structural similarity. (e) Ribbon view of kindlin F0 as in (c) with the six tryptophan residues shown in green and the positive charges surrounding W75 shown in purple. (f) The F0 domain of talin-1 showing the remarkable structural similarity between the two F0 domains.

We have used the revised sequence alignment to design a pet151 His-tagged construct encoding the N-terminal region (residues 1–96) of kindlin-1, that is, the predicted F0 domain. The expressed protein was soluble and stable; it was readily purified and the ^1^H-^15^N HSQC NMR spectrum indicated a stable and well-defined protein fold (Fig. 2a). The signals display remarkable dispersion, probably due in part to the large number of aromatic residues in the domain (6 Trp, 1 Tyr, and 1 Phe). The solution structure was calculated from 2024 distance and 54 dihedral angle restraints determined using ^13^C, ^15^N-labeled protein and is shown in Fig. 2b and c with the statistics in Table 1. The kindlin-1 F0 domain adopts a ubiquitin-like β-grasp fold with a five-stranded twisted β-sheet wrapping around the hydrophobic face of an α-helix (topology β1, β2, α1, β3, β4, α2, β5). The first nine residues are disordered; this N-terminal part of the kindlin sequence has no counterpart in talin and is variable in sequence and length between the three isoforms (Fig. S1). Apart from talin-1 F0 (see below; Goult *et al.*, under revision), the closest structural homologues of kindlin F0 identified by a DALI database search^39^ are ubiquitin (2GBM; *Z*-score = 9.7; r.m.s.d. = 1.5 Å) (Fig. 2d), interferon-induced ubiquitin cross-reactive protein ISG15 (1Z2M; *Z*-score = 8.8; r.m.s.d. = 3.7 Å), and the radixin F1 domain (2D10; *Z*-score = 8.7; r.m.s.d. = 2.4 Å).

**Fig. 3:**
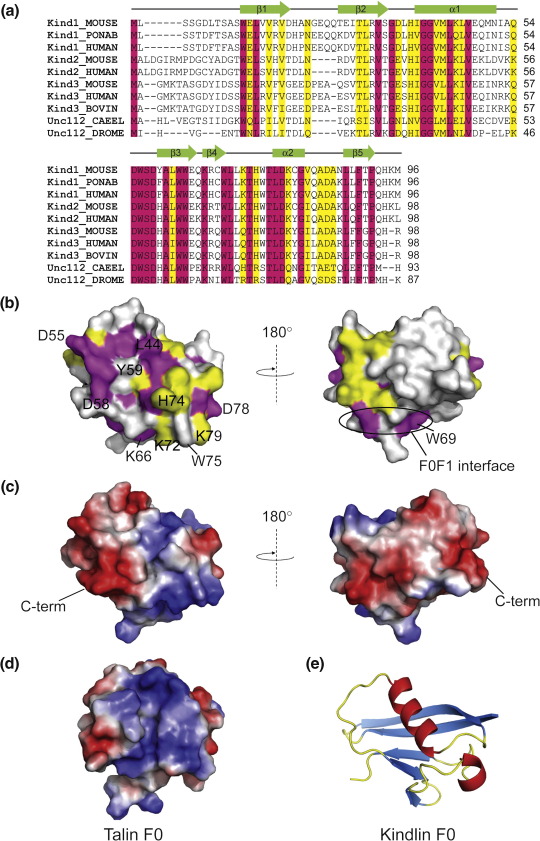
Conservation of the kindlin F0 domain. (a) Sequence alignment of mouse kindlin-1 residues 1–96 with the corresponding regions of other kindlins. Magenta, invariant residues; yellow, residues that are highly conserved. The secondary-structure elements are shown above the alignments. The sequences used are as follows: kindlin-1 of mouse (P59113), orangutan (Q5R8M5), and human (Q9BQL6); kindlin-2 of mouse (Q8CIB5) and human (Q96AC1); kindlin-3 of mouse (Q8K1B8), human (Q86UX7), and cow (Q32LP0); Unc-112 of *C.elegans* (Q18685) and *Drosophila* (Q9VZ13). (b) Surface representation of the kindlin-1 F0 domain showing the position of the conserved residues identified in (a). A conserved surface comprising helix 1 and the loop between helix 1 and strand 3 is present in all kindlin isoforms. (c) Electrostatic surface of kindlin-1 F0. The left panel is oriented as in (b) and (e). The flexible N-terminus (residues 1–11) is not shown. (d) Electrostatic surface of the talin-1 F0 domain—the orientation is the same as that for kindlin-1 F0 shown in the left panels in (b) and (c). (e) Ribbon representation of kindlin-1 F0 showing the orientation of the protein in the left panels in (b) and (c).

The position of conserved residues in kindlin F0 was analyzed using ConSurf^40,41^ and T-Coffee.^42^ The alignments reveal that the domain is well conserved across all kindlins and across all species (Fig. 3a). The conserved regions and electrostatic potential mapped onto the surface representation of kindlin-1 F0 are shown in Fig. 3b and c. The amino acid residues and surface charge are clearly more conserved on one face (left panel) than on the other face of the domain. Three acidic residues (D55, D58, and D78) and one basic residue (K66) are absolutely conserved, but a number of others are conserved in all vertebrate kindlin F0 domains.

Kindlin-1 F0 is unusual in that it has six tryptophan residues (Fig. 2e) in contrast to the single tryptophan in talin-1 F0 (Fig. 2f). Of these six tryptophan residues, W12 and W63 are buried and presumably play a structural role, while the others are at least partly solvent exposed and may be involved in protein–protein interactions. Residues W62 and W69 are on the surface of F0 and occupy equivalent positions to W61 and F50 in talin F0 (Fig. 2e and f and Fig. S5). The structure of the talin F0F1 domain (Goult *et al.*, under revision) shows that these residues are important components of a well-defined, relatively rigid interface between F0 and F1. Therefore, W62 and W69 would be expected to be components of a similar interface between the F0 and F1 domains of kindlin-1. In support of this notion, the residues predicted to be important in the F0F1 interface, including W62 and W69, are conserved in all kindlins and across species (Fig. 3a).

Tryptophan 75 is solvent exposed and is surrounded by positive charges, including K72, H74, and K79 (Fig. 2f). These residues are conserved in all vertebrate kindlins, except for K72, which is a glutamine in kindlin-3. This unusual combination of residues marks this out as a potential binding surface. It is clear from Fig. 3b that one face of the domain is significantly more conserved than the other, and sequence comparisons identify a highly conserved surface involving one face of helix 1 (residues G40, G41, L44, and V47) and the DWSD motif in the loop between helix 1 and β-strand 3; W56 is partially exposed in the middle of this surface. This conserved surface patch may also represent a ligand-binding surface. Interestingly, there is a tyrosine (Y59) or phenylalanine in the middle of the conserved patch in kindlin-1, but a histidine in kindlin-2 and kindlin-3, as well as in the *C. elegans* and *Drosophila* kindlins (Fig. 3b). It is possible that this substitution imparts different binding properties on each of the kindlins.

### Comparison of the F0 domains of talin and kindlin

Despite the rather low sequence identity between the F0 domains of kindlin and talin, the structural similarity between them is high (Fig. S2) with an r.m.s.d. of 1.8 Å for the secondary-structure regions. Figure 3d shows that the talin-1 surface is more basic than that of kindlin-1 and has fewer uncharged residues on its surface. In addition, there are some distinct structural differences between the two domains that may have functional significance. For example, the β3–β4 loop, which is 8 residues long in talin, is just 4 residues in kindlin (Fig. 1b and Fig. S2b and c). By contrast, in talin, the loop between strands β1 and β2 is only 3 residues long, forming a tight hairpin turn, whereas in kindlin-1, it is 9 residues long (Fig. 1b and Fig. S2b and c). The length and sequence of the β1–β2 loop differs significantly between kindlin-1, kindlin-2, and kindlin-3, but it is highly conserved between species within each isoform. A similar situation is found at the N-terminus. In the talin F0 structure, the secondary-structure elements begin at residue 3, but in kindlin-1, there are an additional 11 residues (MLSSGDLTSAS) at the N-terminus, which are unstructured (Fig. 2b), as indicated by the lack of long-range nuclear Overhauser enhancements (NOEs). As for the β1–β2 loop, the length and sequence of this unstructured region vary between the kindlin isoforms, but it is highly conserved within each isoform (Fig. 3a). These characteristics of the N-terminal region and the β1–β2 loop suggest that they may help to define the different functional characteristics of the three kindlin isoforms.

### The kindlin F0 domain is required for integrin activation

**Fig. 4:**
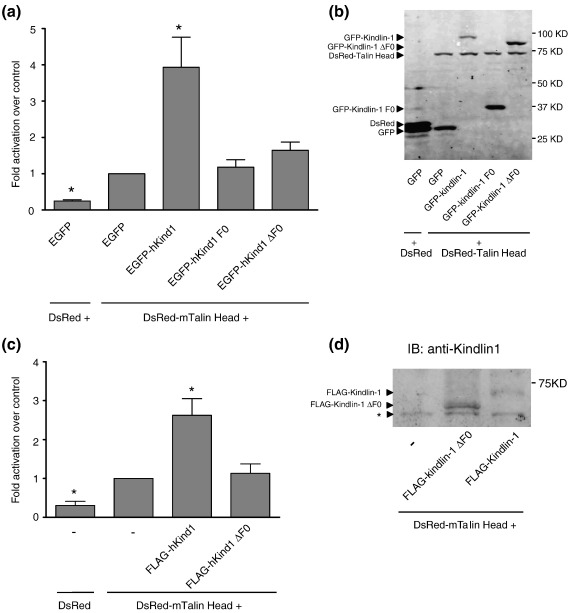
The kindlin F0 domain is important for kindlin-1-mediated αIIbβ3 integrin activation. CHO cells stably expressing αIIbβ3 integrin were co-transfected with DsRed or DsRed-tagged talin head (1–433) and GFP or GFP-tagged kindlin-1 cDNAs as indicated. (a) Activation indices of αIIbβ3 integrin from co-expressing cells with similar fluorescence of GFP and DsRed tags were calculated and normalized for integrin expression (see Materials and Methods). The results represent the means ± standard error (*n* ≥ 3). Results that are significantly different from GFP + DsRed talin head (*p* < 0.05) are indicated (*). (b) Total lysates from double transfected CHO cells were separated by SDS-PAGE and analyzed by immunoblot for tagged proteins using specific anti-GFP (Rockland), anti-DsRed (Santa Cruz), and Alexa 680 Fluor-coupled anti-goat (Invitrogen) antibodies. (c) αIIbβ3-expressing CHO cells were transfected as above but FLAG-tagged kindlin-1 or kindlin-1ΔF0 was substituted for the GFP-tagged versions. Transfected cells were gated based on equal DsRed fluorescence, and activation indices were calculated and normalized for integrin expression as above. Activation indices were expressed relative to DsRed talin alone and represent means ± standard error (*n* ≥ 3). (d) Total cell lysates from cells transfected in (c) were separated by SDS-PAGE and probed for recombinant kindlin proteins. CHO cells do not contain detectable levels of endogenous kindlin-1^43^—the nonspecific band (*) migrates faster than that expected for intact kindlin-1.

We^34,43^ and others^8,25,26^ have previously shown that kindlins are involved in regulating the integrin activation state and specifically that when overexpressed in CHO cells, kindlin-1 and kindlin-2 can cooperate with the talin head to activate αIIbβ3 integrins.^28,29,36,43^ We have also recently shown that in talin, the talin F0 domain is required in addition to the integrin-binding phosphotyrosine-binding-like F3 domain for efficient activation of β1 and β3 integrins.^21^ Given the similarities between the talin and kindlin FERM domains, we tested whether the kindlin F0 domain was also required for the co-activating activity of kindlin-1 on αIIbβ3 integrins. We co-expressed DsRed-tagged talin head along with green fluorescent protein (GFP)-tagged full-length human kindlin-1, the kindlin-1 F0 domain (amino acids 1–96), or kindlin-1 lacking F0 (kindlin-1ΔF0; amino acids 94–677) in αIIbβ3-expressing CHO cells and assessed integrin activation using the activation-specific, ligand-mimetic, anti-αIIbβ3 monoclonal antibody PAC1 in three-color fluorescence-activated cell sorting (FACS) assays as previously described.^43^ Fusion partners and expression levels were selected to optimize cooperative kindlin-1 activation and our data show that, as previously reported, when co-expressed with talin head, full-length kindlin-1 strongly potentiates αIIbβ3 activation (Fig. 4). However, co-expressed kindlin-1 F0 did not increase αIIbβ3 integrin activation compared to the talin head control, and kindlin-1ΔF0 produced only a small, statistically insignificant (*p* = 0.07) increase in αIIbβ3 activation (Fig. 4). Since, in all cases, measurements were made on cells gated to have comparable levels of DsRed talin head and comparable levels of GFP-kindlin-1, GFP-kindlin-1 F0, or GFP-kindlin-1ΔF0, differences in expression of the recombinant proteins cannot account for the inability of kindlin-1 F0 or kindlin-1ΔF0 to co-activate αIIbβ3. Furthermore, in each experiment, we assessed αIIbβ3 expression levels in transfected cells, and PAC1 binding was normalized to this level to exclude any effects due to changes in integrin expression level. Finally, to exclude the possibility that GFP-kindlinΔF0 fails to activate because of steric hindrance by the N-terminal GFP tag, we repeated the experiments using FLAG-tagged kindlin-1 constructs co-expressed with DsRed alone or DsRed talin head. As shown in Fig. 4c, replacing the GFP tag with the short, eight-amino-acid FLAG tag did not alter the results. In summary, our data indicate that the F0 domain of kindlin-1 is necessary, but not sufficient, to mediate the synergistic effect of kindlin-1 with talin on integrin activation.

### The kindlin F0 domain is required for targeting to FAs

**Fig. 5:**
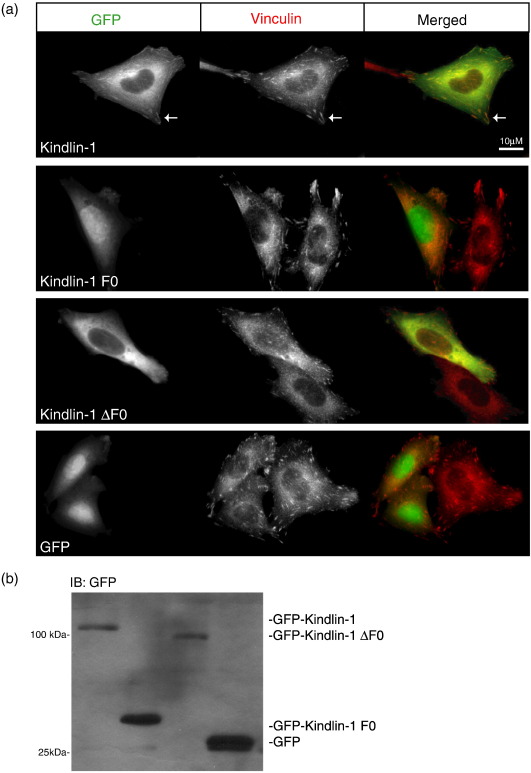
The kindlin-1 F0 domain is required for targeting to FAs. Images of CHO cells stably expressing αIIbβ3 integrin transiently transfected with GFP-tagged kindlin-1 wild-type, F0, and ΔF0 expression constructs after 4 h growth on fibrinogen-coated coverslips. GFP-kindlin-1 co-localizes with endogenous vinculin at FAs (white arrows). Neither GFP-kindlin-1 F0 nor GFP-kindlin-1ΔF0 clusters in vinculin-rich FAs.

Kindlin-1 localizes to FAs, and this localization depends on a functional integrin-binding site in the F3 domain.^34,43^ To investigate whether the F0 domain also plays a role in this process, we compared the localization of GFP-kindlin-1, GFP-kindlin-1-F0, and GFP-kindlin-1ΔF0 in αIIbβ3-expressing CHO cells spreading on fibrinogen. Fibrinogen is a ligand for αIIbβ3, and adhesion, spreading, and formation of FAs by αIIbβ3-expressing CHO cells on fibrinogen are dependent on αIIbβ3.^44^ Cells were transfected with GFP-kindlin expression constructs, and 4 h after plating on fibrinogen-coated coverslips, cells were fixed and stained for vinculin as a marker of FAs. As shown in Fig. 5, cells spread and formed vinculin-containing FAs, but only GFP-kindlin-1 co-localized with vinculin while the GFP-kindlin-1-F0 and GFP-kindlin-1ΔF0 exhibited diffuse cytoplasmic staining. Like GFP alone, GFP-F0 also exhibited nuclear staining presumably due to its small size. Thus, the F0 domain is required but not sufficient for FA targeting of kindlin-1.

It remains to be established how kindlin F0 contributes to talin-mediated integrin activation and the localization of kindlin-1 to FAs. No binding partners for F0 have been identified to date, although both migfilin and ILK have been inferred to bind to the N-terminal region of kindlins.^24^ Migfilin, which is required for integrin-mediated cell spreading,^45^ has recently been reported to act as a molecular switch in integrin activation via its ability to bind filamin.^46,47^ The filamin-binding site on β-integrin tails overlaps that for talin,^46,48,49^ and filamin therefore has the potential to inhibit talin-mediated integrin activation. Indeed, filamin knockdown increases integrin activation in various cell lines.^49,50^ Migfilin has now been shown to competitively inhibit binding of filamin to β-integrin tails and to enhance β1 and β3 integrin activation in cells.^46,47^ Thus, by sequestering filamin, a kindlin/migfilin complex might indirectly increase talin binding to integrin and thereby potentiate integrin activation. The ILK/PINCH/Parvin complex is also known to be important in a variety of integrin-mediated events,^4^ and ILK has recently been shown to cooperate with talin in the inside-out activation of exogenously expressed αIIbβ3 integrin in CHO cells by an as yet unidentified mechanism.^51^ Interestingly, kindlin-2 is required for the localization of ILK to FAs,^29^ and this may also contribute to the synergy between kindlins and talin. Finally, N-terminal deletions of kindlin have been reported to markedly reduce its binding to integrins.^36,43^ However, the constructs used were based on earlier sequence alignments and deleted not only kindlin F0 but also a substantial part of the F1 domain. Therefore, these experiments do not establish a requirement for F0 in the binding of kindlin to integrins. The revised domain structure of kindlin reported here will facilitate further studies on the roles of the individual domains in the structure and function of kindlins.

## Materials and Methods

### Expression of recombinant kindlin polypeptides

The cDNAs encoding murine kindlin-1 residues 1–96 (F0), residues 141–249 (F1 insert), residues 94–275 (F1), and residues 94–275 Δ145–244 (F1 Δinsert) were synthesized by PCR using a mouse kindlin-1 cDNA as template and cloned into the expression vector pet-151TOPO (Invitrogen). Kindlin polypeptides were expressed in *Escherichia* *coli* BL21 STAR (DE3) cultured in either LB for unlabeled protein or M9 minimal media for preparation of isotopically labeled samples for NMR. Recombinant His-tagged kindlin polypeptides were purified by nickel affinity chromatography following standard procedures. The His-tag was removed by cleavage with AcTEV protease (Invitrogen), and the proteins were further purified by anion-exchange chromatography. Protein concentrations were determined using extinction coefficients at 280 nm calculated from the aromatic amino acid content according to ProtParam‡ as follows: kindlin F0: 34,490 M^− 1^ cm^− 1^; kindlin F1: 20,065 M^− 1^ cm^− 1^; kindlin residues 145–244: 11,460 M^− 1^ cm^− 1^; F1 (Δ145–244): 8480 M^− 1^ cm^− 1^.

### NMR spectroscopy

NMR experiments for the resonance assignment and structure determination of kindlin 1–96 were carried out with 0.5 mM protein in 20 mM sodium phosphate, pH 6.5, 50 mM NaCl, 2 mM DTT, and 10% (v/v) ^2^H_2_O. NMR spectra were obtained at 298 K using Bruker AVANCE DRX 600 or AVANCE DRX 800 spectrometers, both equipped with CryoProbes. Proton chemical shifts were referenced to external 2,2-dimethyl-2-silapentane-5-sulfonate, and ^15^N and ^13^C chemical shifts were referenced indirectly using recommended gyromagnetic ratios.^52^ Spectra were processed with TopSpin (Bruker) and analyzed using Analysis.^53^ 3D HNCO, HN(CA)CO, HNCA, HN(CO)CA, HNCACB, and HN(CO)CACB experiments were used for the sequential assignment of the backbone NH, N, CO, C^α^, and C^β^ resonances as described previously.^54^ Side-chain assignments were obtained using 3D HBHA(CO)NH, HBHANH, H(C)CH-total correlated spectroscopy, and (H)CCH-total correlated spectroscopy experiments. Aromatic side-chain assignments were obtained using ^13^C-resolved 3D nuclear Overhauser enhancement spectroscopy (NOESY)-HSQC. The resonance assignments of kindlin 1–96 have been deposited in the Biological Magnetic Resonance Data Bank§ with the accession number 16163.

### Structure calculations

Distance restraints were obtained from the following experiments: 3D ^15^N-edited NOESY-HSQC (800 MHz, 100 ms), ^13^C-edited NOESY-HSQC (800 MHz, 100 ms), ^13^C-edited NOESY-HSQC (800 MHz, 80 ms) on aromatics, and 2D NOESY in 2-->H_2_O (800 MHz, 100 ms). All NOESY peaks were picked semiautomatically in Analysis with noise and artifact peaks removed manually. Cross-peak intensities were used to evaluate target distances. Dihedral restraints (φ/ψ) were obtained from chemical shifts using the TALOS database.^55^ Hydrogen-bond restraints within secondary-structure elements identified from initial rounds of structure calculation were incorporated based on the temperature dependence of amide proton chemical shifts^56^ using a series of ^1^H-^15^N HSQC spectra collected in the range 15 –35 °C. Initial models were generated with CYANA using the CANDID^57^ method for NOESY cross-peak assignment and calibration. These models were used as initial structures in structure calculations by ARIA.^58^ The acceptance tolerances in the standard protocol of ARIA 1.2 were modified to set violation tolerances to 5.0, 2.0, 1.0, 0.5, 2.0, 0.5, and 0.1 Å for iterations 2–8, respectively, with iteration 1 containing the initial models. Any cross peaks rejected by ARIA were checked manually and those found to be reliable were added to the calculation. Two hundred structures were calculated at each iteration; the 20 lowest-energy structures were retained and 10 were used for final restraint analysis. The 30 lowest-energy structures from iteration 8 were further refined in the presence of explicit water molecules. Molecular models were generated using PyMOL.^59^ The structural statistics are presented in Table 1. The set of 20 lowest-energy structures has been submitted to the PDB∥ with the accession number 2KMC.

### Analysis of integrin activation

The activation state of stably overexpressed αIIbβ3 integrin in CHO cells transiently expressing DsRed-tagged mouse talin head and/or GFP-human kindlin-1 fragments was assessed in three-color FACS assays using a modification of previously described methods.^15,21,43,60^ Briefly, αIIbβ3-expressing CHO cells^61^ were co-transfected with the indicated GFP and DsRed expression constructs using Lipofectamine (Invitrogen), and 24 h later, the cells were suspended and incubated with ligand-mimetic anti-αIIbβ3 monoclonal antibody PAC1^15,62–64^ (BD Biosciences) in the presence or absence of 10 mM ethylenediaminetetraacetic acid. αIIbβ3 integrin expression was assessed in parallel by staining with D57 antibody.^61^ Bound PAC1 was detected using Alexa 647 fluorophore-conjugated goat anti-mouse IgM (Invitrogen). Activation of αIIbβ3 in doubly transfected (GFP-positive and Red-positive) cells was quantified in three-color flow cytometric assays, and the αIIbβ3 activation index was defined as AI = (*F* − *F*_o_)/(*F*_integrin_), where *F* is the geometric mean fluorescence intensity (MFI) of PAC1 binding, *F*_o_ is the MFI of PAC1 binding in the presence of ethylenediaminetetraacetic acid, and *F*_integrin_ is the standardized ratio of D57 binding to transfected cells. *F*_integrin_ expression ratio was defined for double expressing cells as follows: *F*_integrin_ = (*F*_trans_)/(*F*_untrans_), where *F*_trans_ is the geometric MFI of D57 binding to double expressing cells and *F*_untrans_ is the MFI of D57 binding to untransfected cells. FACS data analysis was carried out using FlowJo FACS analysis software and statistical analysis using GraphPad Prism.

DsRed-tagged mouse talin head (amino acids 1–433) was generated as previously described^21^ and subcloned into pDsRed-monomer-C1 vector (Clontech Laboratories). Human kindlin-1 fragments were generated by PCR from previously described human kindlin-1 constructs^43^ and cloned into pEGFP-C1 (Clontech Laboratories) or FLAG-CMV2 (Sigma).

### Immunofluorescence

CHO cells stably expressing αIIbβ3 integrin were transiently transfected with 3 μg of indicated cDNAs using Lipofectamine™ (Invitrogen). Twenty-four hours after transfection, cells were detached and allowed to re-adhere and spread on fibrinogen-coated (10 μg/ml) coverslips. After 4 h of plating, cells were fixed, permeabilized, and stained with monoclonal anti-vinculin antibody (Sigma-Aldrich) and anti-mouse Alexa 568 (Invitrogen) as described previously.^46^

### Accession numbers

Coordinates for mouse kindlin-1 F0 have been deposited in the PDB with accession number 2KMC.

## Figures and Tables

**Table 1:** Solution structure determination of kindlin-1 (residues 1–96)

Experimental restraints	
Unique/Ambiguous NOEs	2024/146
Intraresidue	1041/40
Sequential	323/23
Short range (1 < [*i* − *j*] < 5)	183/28
Long range ([*i* − *j*] > 4)	477/55
φ/ψ dihedral angles^a^	54
Energies (kcal mol^− 1^)^b^	
Total	− 3808.1 ± 50.4
van der Waals	− 889.2 ± 12.5
NOE	11.49 ± 3.02
r.m.s.d.^b^	
NOEs (Å) (no violations > 0.5 Å)	0.011 ± 0.001
Dihedral restraints (°) (no violations > 0.5°)	0.30 ± 0.09
Bonds (Å)	0.0035 ± 0.0001
Angles (°)	0.47 ± 0.02
Impropers (°)	1.30 ± 0.08
Ramachandran map analysis (%)^c^	
Allowed regions	85.9
Additional allowed regions	12.8
Generously allowed regions	0.7
Disallowed regions	0.5
Pairwise r.m.s. difference (Å)^d^	
Residues 10–96	0.73 (1.20)
Secondary structure	0.35 (0.81)

a From chemical shifts using TALOS.

b Calculated in ARIA 1.2 for the 20 lowest-energy structures refined in water.

c Obtained using PROCHECK-NMR.^38^

d For backbone atoms; values for all heavy atoms are in parentheses.

## Appendix A

Fig. S1 . Amino acid sequence alignments of the three mouse kindlins; kindlin-1 (P59113), kindlin -2 (Q8CIB5), and kindlin-3 (Q8K1B8). The F1 insert is boxed. An NPxY motif located in the F1–F2 linker region in kindlin-1 and kindlin-2 (not present in kindlin-3) is colored red. Fig. S2. (a) Sequence alignment of the F0 domain of mouse kindlin-1 and talin-1. The secondary structure is shown above (kindlin) and below (talin) the alignment. While the primary sequence shows only 13% identity and 37% similarity, the secondary structure is highly conserved. Magenta indicates invariant residues; yellow indicates residues that are highly conserved. (b and c) Ribbon view of the F0 subdomains of kindlin-1 (b) and talin-1 (c) showing the similarities and differences between the two domains. Fig. S3. (a) SDS-PAGE gel of the kindlin-1 F1 insert (residues 145–244). (b) 1-->H–15-->N HSQC spectrum of the F1 insert shows that it is predominantly unstructured as indicated by the poor signal dispersion, although some weaker signals may suggest some ordered elements. (c) Sequence alignment of the F1 inserts of talin-1 and kindlin-1 using T-Coffee^42^ (d) Secondary-structure prediction for the F1 insert of kindlin-1 calculated using PSIPRED.^65,66^ 65. Bryson, K., McGuffin, L. J., Marsden, R. L., Ward, J. J., Sodhi, J. S. & Jones, D. T. (2005). Protein structure prediction servers at University College London. *Nucleic Acids Res.***33**, W36–W38. 66. Jones, D. T. (1999). Protein secondary structure prediction based on position-specific scoring matrices. *J. Mol. Biol.***292**, 195–202. Fig. S4. The F0F1 interface in talin (gray) (Goult *et al.*, under revision; PDB ID: 2KMA), showing the position of W61 and F50. Superimposed in green is the structure of kindlin-1 F0 domain, showing the positions of W62 and W69.

## Footnotes

† http://www.cellmigration.org/resource/proteomics

‡ www.expasy.org

§ http.//www.bmrb.wisc.edu

∥ www.rcsb.org
