# Supplementary material for: The Structure of the N-Terminus of Kindlin-1: A Domain Important for αIIbβ3 Integrin Activation
Source: J Mol Biol. 2009 Dec 18;394(5):944–56. doi: 10.1016/j.jmb.2009.09.061 (PMC2963925; doi:10.1016/j.jmb.2009.09.061)
Supplement: Fig. S1 — . Amino acid sequence alignments of the three mouse kindlins; kindlin-1 (P59113), kindlin -2 (Q8CIB5), and kindlin-3 (Q8K1B8). The F1 insert is boxed. An NPxY motif located in the F1–F2 linker region in kindlin-1 and kindlin-2 (not present in kindlin-3) is colored red. Fig. S2. (a) Sequence alignment of the F0 domain of mouse kindlin-1 and talin-1. The secondary structure is shown above (kindlin) and below (talin) the alignment. While the primary sequence shows only 13% identity and 37% similarity, the secondary structure is highly conserved. Magenta indicates invariant residues; yellow indicates residues that are highly conserved. (b and c) Ribbon view of the F0 subdomains of kindlin-1 (b) and talin-1 (c) showing the similarities and differences between the two domains. Fig. S3. (a) SDS-PAGE gel of the kindlin-1 F1 insert (residues 145–244). (b) 1-->H–15-->N HSQC spectrum of the F1 insert shows that it is predominantly unstructured as indicated by the poor signal dispersion, although some weaker signals may suggest some ordered elements. (c) Sequence alignment of the F1 inserts of talin-1 and kindlin-1 using T-Coffee42 (d) Secondary-structure prediction for the F1 insert of kindlin-1 calculated using PSIPRED.65,66 65. Bryson, K., McGuffin, L. J., Marsden, R. L., Ward, J. J., Sodhi, J. S. & Jones, D. T. (2005). Protein structure prediction servers at University College London. Nucleic Acids Res.33, W36–W38. 66. Jones, D. T. (1999). Protein secondary structure prediction based on position-specific scoring matrices. J. Mol. Biol.292, 195–202. Fig. S4. The F0F1 interface in talin (gray) (Goult et al., under revision; PDB ID: 2KMA), showing the position of W61 and F50. Superimposed in green is the structure of kindlin-1 F0 domain, showing the positions of W62 and W69. [file mmc1.ppt]

## Slide 1
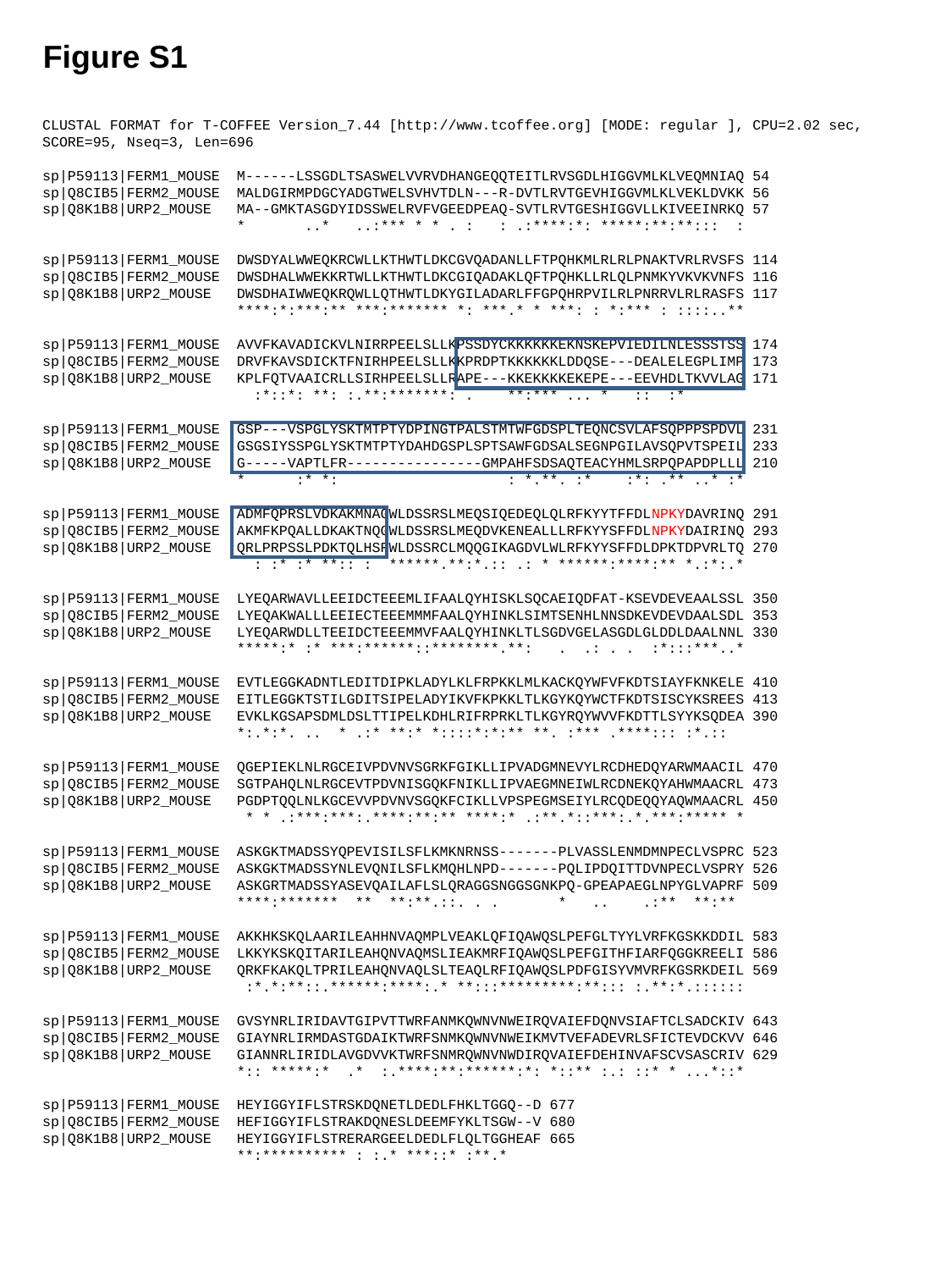

Figure S1
CLUSTAL FORMAT for T-COFFEE Version_7.44 [http://www.tcoffee.org] [MODE: regular ], CPU=2.02 sec,
SCORE=95, Nseq=3, Len=696
sp|P59113|FERM1_MOUSE M------LSSGDLTSASWELVVRVDHANGEQQTEITLRVSGDLHIGGVMLKLVEQMNIAQ 54
sp|Q8CIB5|FERM2_MOUSE MALDGIRMPDGCYADGTWELSVHVTDLN---R-DVTLRVTGEVHIGGVMLKLVEKLDVKK 56
sp|Q8K1B8|URP2_MOUSE MA--GMKTASGDYIDSSWELRVFVGEEDPEAQ-SVTLRVTGESHIGGVLLKIVEEINRKQ 57
 * ..* ..:*** * * . : : .:****:*: *****:**:**::: :
sp|P59113|FERM1_MOUSE DWSDYALWWEQKRCWLLKTHWTLDKCGVQADANLLFTPQHKMLRLRLPNAKTVRLRVSFS 114
sp|Q8CIB5|FERM2_MOUSE DWSDHALWWEKKRTWLLKTHWTLDKCGIQADAKLQFTPQHKLLRLQLPNMKYVKVKVNFS 116
sp|Q8K1B8|URP2_MOUSE DWSDHAIWWEQKRQWLLQTHWTLDKYGILADARLFFGPQHRPVILRLPNRRVLRLRASFS 117
 ****:*:***:** ***:******* *: ***.* * ***: : *:*** : ::::..**
sp|P59113|FERM1_MOUSE AVVFKAVADICKVLNIRRPEELSLLKPSSDYCKKKKKKEKNSKEPVIEDILNLESSSTSS 174
sp|Q8CIB5|FERM2_MOUSE DRVFKAVSDICKTFNIRHPEELSLLKKPRDPTKKKKKKLDDQSE---DEALELEGPLIMP 173
sp|Q8K1B8|URP2_MOUSE KPLFQTVAAICRLLSIRHPEELSLLRAPE---KKEKKKKEKEPE---EEVHDLTKVVLAG 171
 :*::*: **: :.**:*******: . **:*** ... * :: :*
sp|P59113|FERM1_MOUSE GSP---VSPGLYSKTMTPTYDPINGTPALSTMTWFGDSPLTEQNCSVLAFSQPPPSPDVL 231
sp|Q8CIB5|FERM2_MOUSE GSGSIYSSPGLYSKTMTPTYDAHDGSPLSPTSAWFGDSALSEGNPGILAVSQPVTSPEIL 233
sp|Q8K1B8|URP2_MOUSE G-----VAPTLFR----------------GMPAHFSDSAQTEACYHMLSRPQPAPDPLLL 210
 * :* *: : *.**. :* :*: .** ..* :*
sp|P59113|FERM1_MOUSE ADMFQPRSLVDKAKMNAGWLDSSRSLMEQSIQEDEQLQLRFKYYTFFDLNPKYDAVRINQ 291
sp|Q8CIB5|FERM2_MOUSE AKMFKPQALLDKAKTNQGWLDSSRSLMEQDVKENEALLLRFKYYSFFDLNPKYDAIRINQ 293
sp|Q8K1B8|URP2_MOUSE QRLPRPSSLPDKTQLHSRWLDSSRCLMQQGIKAGDVLWLRFKYYSFFDLDPKTDPVRLTQ 270
 : :* :* **:: : ******.**:*.:: .: * ******:****:** *.:*:.*
sp|P59113|FERM1_MOUSE LYEQARWAVLLEEIDCTEEEMLIFAALQYHISKLSQCAEIQDFAT-KSEVDEVEAALSSL 350
sp|Q8CIB5|FERM2_MOUSE LYEQAKWALLLEEIECTEEEMMMFAALQYHINKLSIMTSENHLNNSDKEVDEVDAALSDL 353
sp|Q8K1B8|URP2_MOUSE LYEQARWDLLTEEIDCTEEEMMVFAALQYHINKLTLSGDVGELASGDLGLDDLDAALNNL 330
 *****:* :* ***:******::********.**: . .: . . :*:::***..*
sp|P59113|FERM1_MOUSE EVTLEGGKADNTLEDITDIPKLADYLKLFRPKKLMLKACKQYWFVFKDTSIAYFKNKELE 410
sp|Q8CIB5|FERM2_MOUSE EITLEGGKTSTILGDITSIPELADYIKVFKPKKLTLKGYKQYWCTFKDTSISCYKSREES 413
sp|Q8K1B8|URP2_MOUSE EVKLKGSAPSDMLDSLTTIPELKDHLRIFRPRKLTLKGYRQYWVVFKDTTLSYYKSQDEA 390
 *:.*:*. .. * .:* **:* *::::*:*:** **. :*** .****::: :*.::
sp|P59113|FERM1_MOUSE QGEPIEKLNLRGCEIVPDVNVSGRKFGIKLLIPVADGMNEVYLRCDHEDQYARWMAACIL 470
sp|Q8CIB5|FERM2_MOUSE SGTPAHQLNLRGCEVTPDVNISGQKFNIKLLIPVAEGMNEIWLRCDNEKQYAHWMAACRL 473
sp|Q8K1B8|URP2_MOUSE PGDPTQQLNLKGCEVVPDVNVSGQKFCIKLLVPSPEGMSEIYLRCQDEQQYAQWMAACRL 450
 * * .:***:***:.****:**:** ****:* .:**.*::***:.*.***:***** *
sp|P59113|FERM1_MOUSE ASKGKTMADSSYQPEVISILSFLKMKNRNSS-------PLVASSLENMDMNPECLVSPRC 523
sp|Q8CIB5|FERM2_MOUSE ASKGKTMADSSYNLEVQNILSFLKMQHLNPD-------PQLIPDQITTDVNPECLVSPRY 526
sp|Q8K1B8|URP2_MOUSE ASKGRTMADSSYASEVQAILAFLSLQRAGGSNGGSGNKPQ-GPEAPAEGLNPYGLVAPRF 509
 ****:******* ** **:**.::. . . * .. .:** **:**
sp|P59113|FERM1_MOUSE AKKHKSKQLAARILEAHHNVAQMPLVEAKLQFIQAWQSLPEFGLTYYLVRFKGSKKDDIL 583
sp|Q8CIB5|FERM2_MOUSE LKKYKSKQITARILEAHQNVAQMSLIEAKMRFIQAWQSLPEFGITHFIARFQGGKREELI 586
sp|Q8K1B8|URP2_MOUSE QRKFKAKQLTPRILEAHQNVAQLSLTEAQLRFIQAWQSLPDFGISYVMVRFKGSRKDEIL 569
 :*.*:**::.******:****:.* **:::*********:**::: :.**:*.::::::
sp|P59113|FERM1_MOUSE GVSYNRLIRIDAVTGIPVTTWRFANMKQWNVNWEIRQVAIEFDQNVSIAFTCLSADCKIV 643
sp|Q8CIB5|FERM2_MOUSE GIAYNRLIRMDASTGDAIKTWRFSNMKQWNVNWEIKMVTVEFADEVRLSFICTEVDCKVV 646
sp|Q8K1B8|URP2_MOUSE GIANNRLIRIDLAVGDVVKTWRFSNMRQWNVNWDIRQVAIEFDEHINVAFSCVSASCRIV 629
 *:: *****:* .* :.****:**:******:*: *::** :.: ::* * ...*::*
sp|P59113|FERM1_MOUSE HEYIGGYIFLSTRSKDQNETLDEDLFHKLTGGQ--D 677
sp|Q8CIB5|FERM2_MOUSE HEFIGGYIFLSTRAKDQNESLDEEMFYKLTSGW--V 680
sp|Q8K1B8|URP2_MOUSE HEYIGGYIFLSTRERARGEELDEDLFLQLTGGHEAF 665
 **:********** : :.* ***::* :**.*

## Slide 2
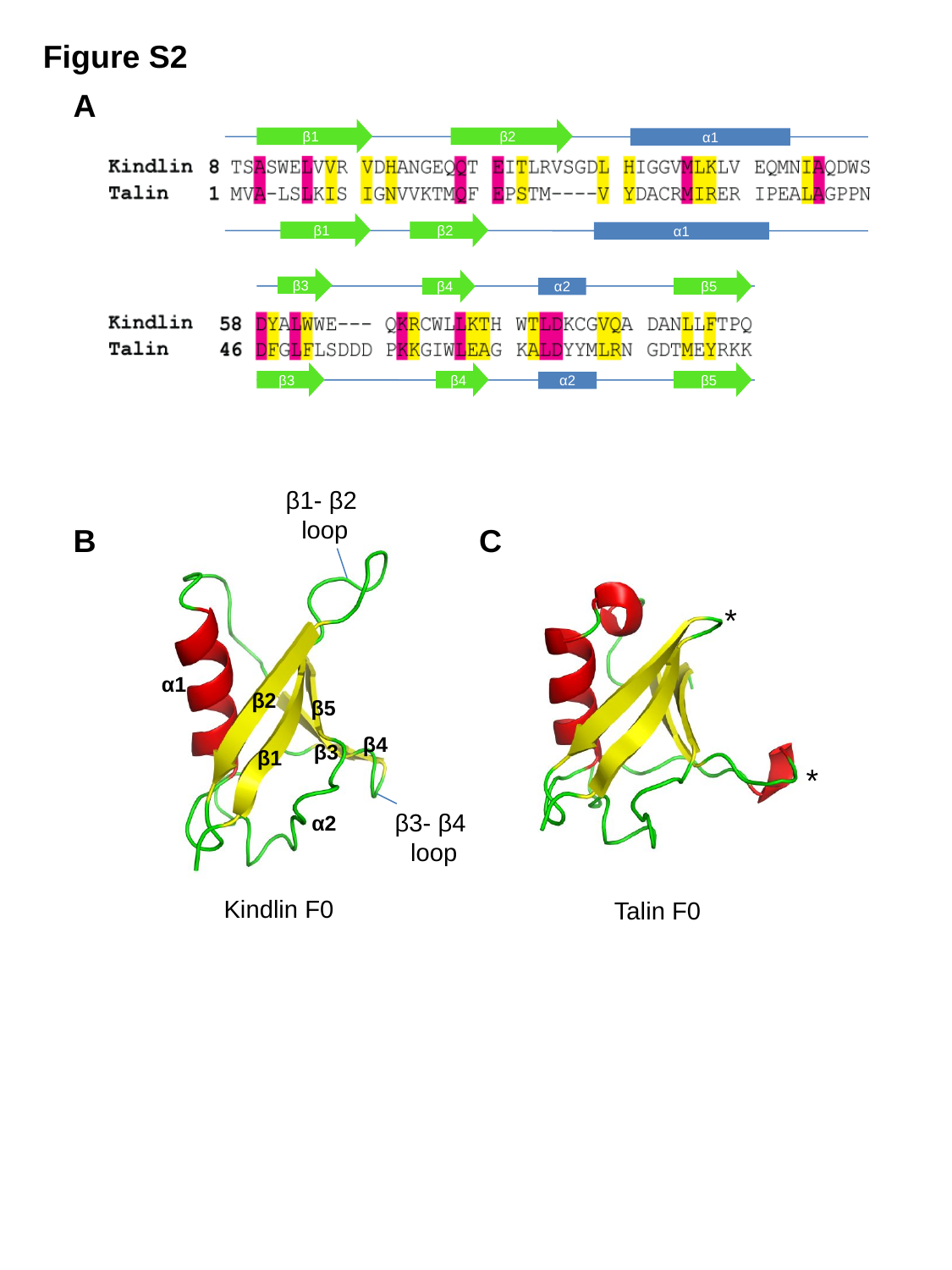

Figure S2
A
β1
β2
α1
β4
β5
α2
β1
β2
α1
β3
β4
β5
α2
β3
β1- β2
loop
B
C
*
α1
β2
β5
β4
β3
β1
*
β3- β4
loop
α2
Kindlin F0
Talin F0
Talin F0

## Slide 3
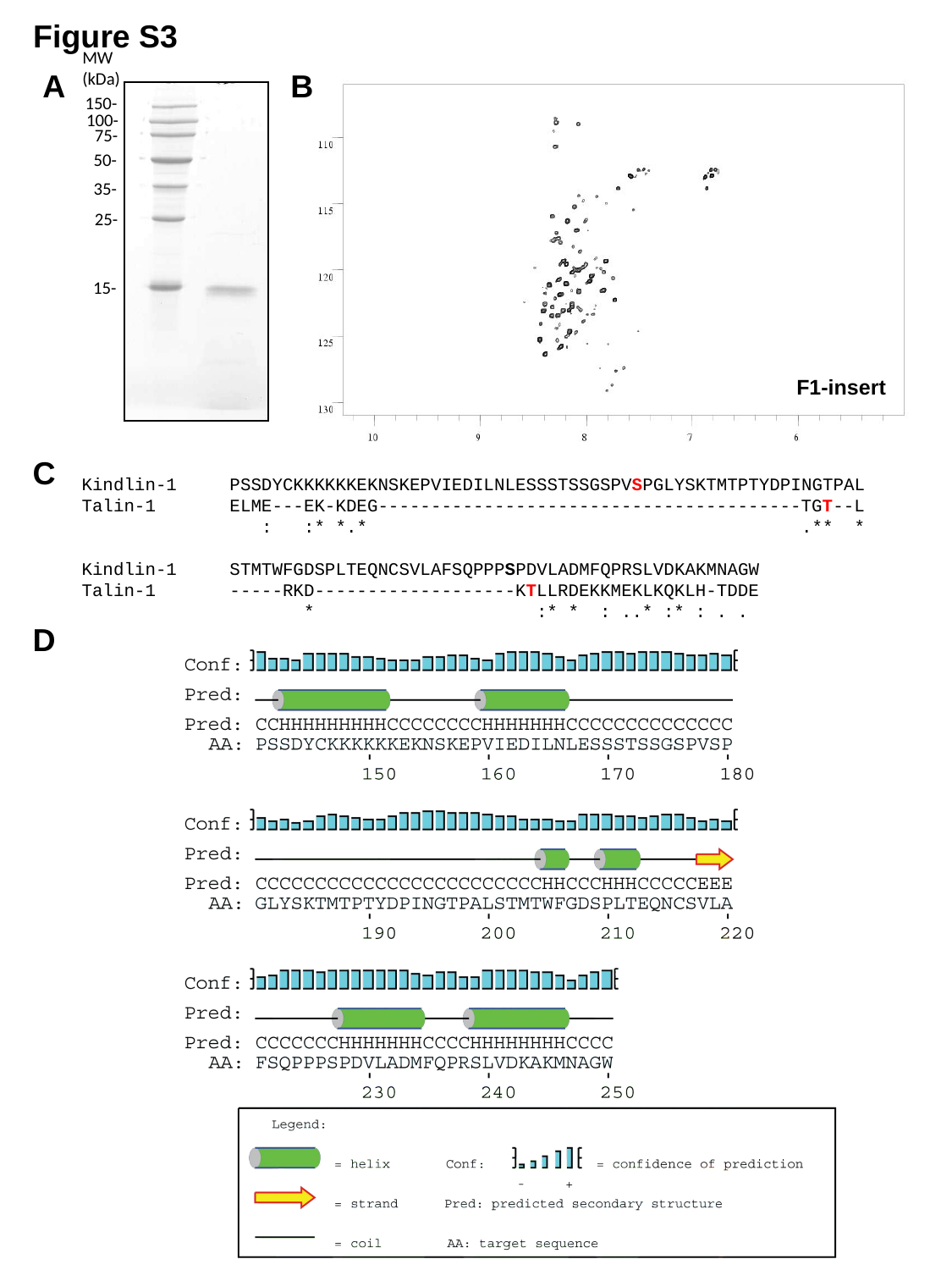

Figure S3
MW
(kDa)
150-
100-
75-
50-
35-
25-
15-
A
B
F1-insert
C
Kindlin-1 PSSDYCKKKKKKEKNSKEPVIEDILNLESSSTSSGSPVSPGLYSKTMTPTYDPINGTPAL
Talin-1 ELME---EK-KDEG----------------------------------------TGT--L
 : :* *.* .** *
Kindlin-1 STMTWFGDSPLTEQNCSVLAFSQPPPSPDVLADMFQPRSLVDKAKMNAGW
Talin-1 -----RKD-------------------KTLLRDEKKMEKLKQKLH-TDDE
 * :* * : ..* :* : . .
D

## Slide 4
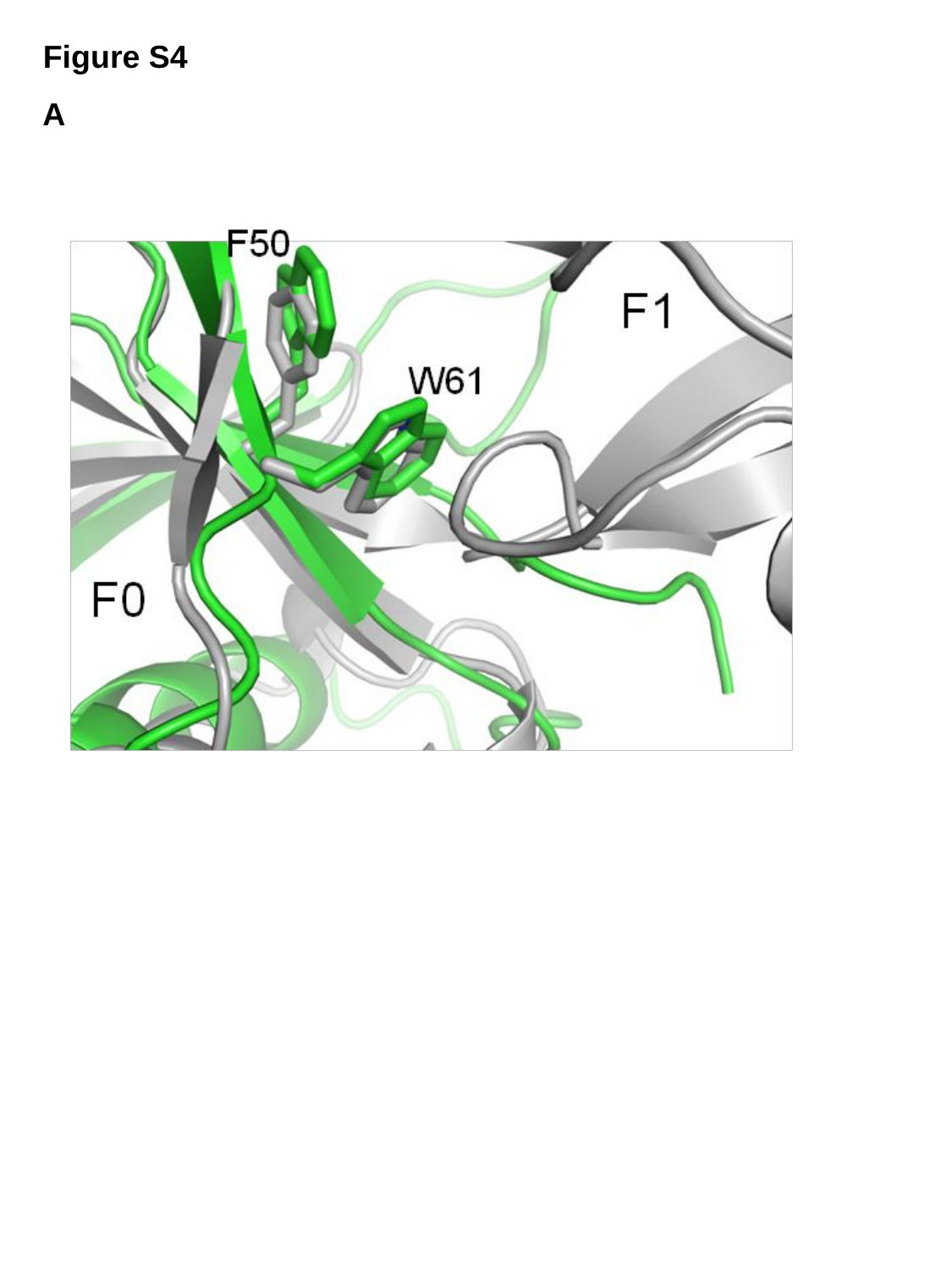

Figure S4
A
